# Factors Associated with Willingness to Pay for Cost-Sharing under Universal Health Coverage Scheme in Yogyakarta, Indonesia: A Cross-Sectional Survey

**DOI:** 10.3390/ijerph192215017

**Published:** 2022-11-15

**Authors:** Diesty Anita Nugraheni, Satibi Satibi, Susi Ari Kristina, Diah Ayu Puspandari

**Affiliations:** 1 Doctoral Graduate Program, Faculty of Pharmacy, Universitas Gadjah Mada, Yogyakarta 55281, Indonesia; 2 Department of Pharmacy, Faculty of Mathematics and Natural Sciences, Universitas Islam Indonesia, Yogyakarta 55584, Indonesia; 3 Department of Pharmaceutics, Faculty of Pharmacy, Universitas Gadjah Mada, Yogyakarta 55281, Indonesia; 4 Department of Health Policy and Management, Faculty of Medicine, Public Health, and Nursing, Universitas Gadjah Mada, Yogyakarta 55281, Indonesia

**Keywords:** willingness to pay, cost-sharing, catastrophic, associated factors, National Health Insurance

## Abstract

Background: National Health Insurance (NHI) in Indonesia requires an appropriate cost-sharing policy, particularly for diseases that require the largest financing. This study examined factors that influence willingness to pay (WTP) for cost-sharing under the universal health coverage scheme among patients with catastrophic illnesses in Yogyakarta, Indonesia. Methods: This was a cross-sectional study using structured questionnaires through direct interviews. The factors related to the WTP for cost-sharing under the NHI scheme in Indonesia were identified by a bivariable logistic regression analysis. Results: Two out of every five (41.2%) participants had willingness to pay for cost-sharing. Sex [AOR = 0.69 (0.51, 0.92)], education [AOR = 1.54 (0.67, 3.55)], family size [AOR = 1.71 (1.07, 2.73)], occupation [AOR = 1.35 (0.88, 2.07)], individual income [AOR = 1.50 (0.87, 2.61)], household income [AOR = 1.47 (0.90, 2.39)], place of treatment [AOR = 2.54 (1.44, 4.45)], a health insurance plan [AOR = 1.22 (0.87, 1.71)], and whether someone receives an inpatient or outpatient service [AOR = 0.23 (0.10, 0.51)] were found to affect the WTP for a cost-sharing scheme with *p* < 0.05. Conclusion: Healthcare (place of treatment, health insurance plan, and whether someone receives an inpatient or outpatient service) and individual socioeconomic (sex, educational, family size, occupational, income) factors were significantly related to the WTP for cost-sharing.

## 1. Introduction

One of the problems encountered by many low- and middle-income countries is financial constraints to provide effective healthcare insurance for their citizens [1]. These countries suffer from the financial burden of catastrophic illnesses due to out-of-pocket expenses, which account for 30% to 85% of the total healthcare costs [2,3]. Many households in these countries use various resources to cover healthcare costs, including savings or borrowing; some of them even have to sell assets or livestock [4,5,6].

The Indonesian government launched the National Health Insurance program (NHI) in 2014 and the Health Security Agency (BPJS-Kes) was appointed to organize this program. The government deemed that the NHI program should have covered all the Indonesian citizens by 2019 according to the Universal Health Coverage (UHC) concept through BPJS-Kes [7]. UHC is defined as a health system that provides a guarantee that each and every citizen of a population can have fair access to high-quality health services, as stated in the law concerning the National Social Security System (SJSN) and implemented by the Social Security Agency (BPJS) [8]. However, until 2019, the coverage of NHI-KIS only reached 83.86% [9]. Along with an increasing number of memberships since 2014, the utilization of the NHI in primary care (FKTP), as well as in the outpatient and inpatient services in secondary care (FKTL), also increased from 2014 to 2019. Most utilizations were conducted in the primary care setting. In 2020, however, a different trend was found, i.e., the number of primary care visits continued to increase to 283.9 million patients, but the number of outpatient and inpatient visits in secondary care decreased compared to that in the previous year. This certainly affected financing, especially for catastrophic illnesses in secondary care [9].

In the period 2014–2018, the NHI scheme organized by BPJS-Kes experienced deficient financing. According to data from the Ministry of Finance from 2014 to 2018, BPJS-Kes experienced a deficit of IDR 1.94 trillion, IDR 4.42 trillion, IDR 6.7 trillion, IDR 13.8 trillion, and IDR 8.86 trillion, respectively (Indonesian rupiah, IDR). Although the cash flow of BPJS-Kes in 2019 had a surplus of IDR 3.29 trillion, it still experienced an asset deficit [9,10]. There are many factors suspected to have caused the BPJS-Kes deficit—for example, the NHI premiums not meeting the actual values, membership arrears, unlimited NHI benefits packages without cost-sharing, and high costs of healthcare services for catastrophic illnesses. It is important to alter the NHI policies to create a health financing mechanism that is better and more beneficial for its members while not causing any losses to BPJS-Kes providers, to guarantee the sustainability of the NHI program.

Some of the factors that potentially cause BPJS-Kes deficits are low premiums, poor management, and fraud. There are many healthcare fraud cases in BPJS-Kes, which are basically due to the weak monitoring system [11]. Furthermore, the BPJS-Kes deficit has an adverse impact on the health management system, health equipment, and pharmacy services. These NHI financing issues require cost and quality control efforts in healthcare services. One of the policies to control quality and costs, as well as detect abuse or moral hazards in the NHI management, is cost-sharing [12]. Cost-sharing is a technique for controlling healthcare costs that requires patients to partially pay for healthcare services [13]. To improve the quality and maintain the sustainability of the health insurance program, providing a reference in the implementation of cost-sharing as part of the efforts to control quality and costs, as well as prevent abuse or moral hazards in health services, is important [12]. Cost-sharing should be implemented in healthcare services that are prone to the misuse of services in the NHI-KIS program. The NHI members or their families must provide confirmation of their willingness to pay for the cost-sharing prior to receiving the healthcare services. The amount of cost-sharing should differ between outpatient and inpatient services [12,14]. However, until recently, there have been no clear guidelines from the Indonesian government concerning the types of health services in which cost-sharing should be applied.

Catastrophic illnesses are a category of illness that has a significant effect on healthcare spending [15]. Until 2021, there were eight catastrophic illnesses covered by the BPJS-Kes financing; the sequence from the largest to the lowest amount of financing is heart disease, cancer, stroke, kidney disease, thalassemia, hemophilia, leukemia, and cirrhosis [16]. In fact, catastrophic illnesses require the highest amount of spending under the NHI services [17]. Insurance can reduce the uncertainty and exposure of an individual to high costs related to catastrophic illnesses [18]. Based on the data on the healthcare cost of insurance in Indonesia from 2016 to 2020, approximately IDR 374.86 billion, 83.31%, was spent on referred services in which catastrophic illnesses are one of the illness types covered by the NHI-KIS program. A catastrophic illness requires long-term and high-cost medical treatment. There were 19.2 million catastrophic illness cases in 2018, with financing of IDR 20.4 trillion or 25% of the total healthcare costs. The largest proportion of the costs was to cover cardiovascular diseases, such as heart disease and circulatory diseases. BPJS-Kes spent up to IDR 10.3 trillion for heart disease claims, IDR 2.5 trillion for stroke claims, and IDR 3.5 trillion for cancer claims [9]. This study focused on these three illnesses, which required the highest amount of NHI financing; catastrophic illnesses still rank first in the list of the health financing covered by the NHI-KIS program. The high financing for catastrophic illnesses indicates that the members gain protection from financial risks. Currently, the government, through the NHI-KIS program, provides protection for its members to be able to access healthcare services despite the high healthcare costs without cost-sharing [15,19], in contrast with the financial risks experienced by the government due to these catastrophic illnesses.

The implementation of cost-sharing will bring some benefits, some of which are to lower drug expenditure because the utilization of healthcare services decreases, to shift some of the provincial government’s financial responsibility to the insurance members/community, to promote a more appropriate pattern of drug use and minimize inappropriate drug use, and to improve technical efficiency in the drug market by improving the awareness of the insurance members/community about drug uses [20]. However, it is necessary to propose solutions that prevent the government from experiencing a financial burden due to high healthcare costs and overutilization [21]. Overutilization cases in the curative and rehabilitative stages of catastrophic illnesses can be prevented by cost-sharing if the community members display readiness to take promotive and preventive measures for catastrophic illnesses, allowing for a decrease in catastrophic cases, which then prevents the application of cost-sharing in the curative and rehabilitative stages.

When a member of the NHI is sick and seeks treatment, the member has to seek treatment in accordance with the BPJS-Kes procedures in order for the healthcare costs to be covered by BPJS-Kes. The procedure for first diagnosis is as follows: the patient should seek treatment in a primary care facility (*Puskesmas* or *Pratama* Clinic); if the disease is included as a catastrophic illness that cannot be treated at primary care, the patient will then be referred to secondary care (Hospital or Main Clinic), which has a collaboration with BPJS-Kes. In general, patients with catastrophic illnesses are referred to secondary care for the first diagnosis, but for the subsequent treatments, they can directly visit the secondary care hospital. Through BPJS-Kes, the government should also cover early detection measures, particularly for illnesses for which the treatments at advanced stage are costly, including cancer. Unfortunately, not all early detection procedures are covered by BPJS-Kes because this insurance only covers the cost if its members are sick. Meanwhile, advanced cancer requires more expensive medicine and treatment, which certainly will affect BPJS-Kes financing. In addition to promoting the early detection program, the government and BPJS-Kes should be willing to implement cost-sharing with private insurance, so that the private insurance will cover any medicines that are not covered by BPJS-Kes. Otherwise, the government and BPJS-Kes will find it difficult to manage the NHI-KIS program by themselves [15]. However, by providing good healthcare services, it is possible that these patients can remain employed, thereby helping the country through productivity and paying taxes, thereby reducing the burden on the healthcare system. If someone develops a catastrophic illness, they might never be able to work again, in which case co-payment is not an option, to a certain extent.

A study by Salampessy et al. [22] showed that, in general, two designs proposed by policy makers affected the complexity of cost-sharing programs, namely the timing and amount of cost-sharing payments. Therefore, it is highly important to collect information concerning the amount that people are willing to pay for a health insurance program [23].

In an effort to obtain a better understanding of the successful design of a cost-sharing program for NHI, a quantitative study was conducted in three hospitals with different levels of care in Yogyakarta, Indonesia. A questionnaire on the willingness to pay for cost-sharing under the NHI scheme was used to determine the demographic status that affected the health expenditure patterns of the population, in Yogyakarta, such as health-seeking behavior, the characteristics and patterns of health expenses, and the willingness to pay for cost-sharing under the NHI scheme. Age, income, level of education, family size, chronic diseases, healthcare needs, types of insurance, utilization of healthcare, types of healthcare facility, awareness of schemes, medical history, and wealth are factors that affect willingness to pay or out-of-pocket expenses, as shown in several studies [3,24,25].

However, there has been limited information about the preferences of the population for a healthcare financing system in a country. Therefore, a study using the contingent valuation to examine the willingness to pay for a health insurance plan set by the government could help to design a cost-sharing policy and to create a good healthcare system [2,26].

Previous studies focus on the willingness to pay for social health insurance or community-based health insurance [27,28,29,30,31,32,33,34,35], factors influencing the willingness to pay for health insurance in general [36,37,38,39,40], and factors that affect the deficits in financing faced by BPJS-Kes as the agency that finances the NHI in Indonesia [10,11,41,42]. This study aimed to assess the factors that affect the willingness to pay for cost-sharing under the NHI scheme among patients with catastrophic illnesses in Yogyakarta, Indonesia.

## 2. Materials and Methods

### 2.1. Design, Setting, and Period

This was a cross-sectional study that was conducted in some hospitals in Yogyakarta, Indonesia. The total population of this province is 3,677,446 people (1,820,400 males and 1,857,046 females) [43], divided into five regencies, 78 subdistricts, 169 urban areas, and 269 rural areas [44]. In 2021, the NHI coverage in Indonesia was 86.56% [16,45], while the coverage in this province was 91.49% [46]. The criterion of this study was that the patient had Contributory Health Insurance in the NHI program. Non-Contributory Health Insurance is a type of health insurance in which the premiums are paid by the government, while Contributory Health Insurance is a type of health insurance in which the premiums are paid by the members, either by paying certain amount of money (for those who do not earn a monthly salary) or by deducting from the monthly salary (for those who earn a monthly salary). There were 1,996,826 (59.4%) people with Non-Contributory Health Insurance and 1,363,553 (40.6%) people with Contributory Health Insurance in Yogyakarta. In addition, at the national level, the figure was similar, i.e., 140,411,958 (59.6%) people with Non-Contributory Health Insurance and 95,307,304 (40.4%) people with Contributory Health Insurance [45].

The study was carried out in three hospitals in Yogyakarta that provide healthcare services for NHI members. The hospitals were categorized as class A and B, i.e., referral hospitals to provide healthcare services, especially for diseases that cannot be treated in primary healthcare. Yogyakarta was selected as the study location instead of other provinces in Indonesia because it had the highest level of universal health coverage in Indonesia, reaching 97.24%. In addition, this province also ranked first for the prevalence of catastrophic illnesses, particularly cancer (the highest in Indonesia), while the prevalence of heart diseases and stroke was the second highest in Indonesia [47].

The study was conducted from 11 September to 30 October 2021 after the study protocol was approved by the Research Ethics Commission of Faculty of Medical Sciences, Universitas Gadjah Mada, Yogyakarta, Indonesia (approval code: KE/FK/0539/EC/2021). All the research participants signed a written informed consent form after the purposes and benefits of the study had been explained to them. The participants were asked to fill out a questionnaire and interviewed in a separate area. We confirmed that this study complied with the WHO-CIOMS 2016.

### 2.2. Population and Eligibility Criteria

The sampling was done using a convenience sampling technique. The exclusion and inclusion criteria were as follows.

#### 2.2.1. Inclusion Criteria Patients Who Were Active Members of the NHI

- Either inpatients or outpatients who were diagnosed with heart diseases, cancer, or stroke;
- Patients who received healthcare services with prescriptions;
- Patients who had undergone treatment at least once using NHI.

#### 2.2.2. Exclusion Criteria

- NHI members with Non-Contributory Health Insurance;
- Patients who did not receive prescription drugs for the current examination;
- BPJS-Kes employees;
- Those who did not fill out all the question items in the questionnaires.

### 2.3. Sample Size and Sampling Procedures

The study on the willingness to pay for cost-sharing under the NHI scheme had a minimum sample size, which was computed based on the following assumptions: 95% confidence level, 5% margin of error, 50.0% proportion of disease prevalence (maximal assumption). Each of the respondents who was diagnosed with heart diseases, cancer, or stroke was calculated using the following formula:

$$n=\frac{Z_{1-\alpha /2\;}^{2}pq}{d^{2}}=\frac{1.96_{}^{2}\times 0.5\times 0.5}{0.05^{2}}=0.96/0.0025=384.16\;(\mathrm{for}\;\mathrm{each}\;\mathrm{diagnosis})$$

where n = minimum sample size for each diagnosis; α = confidence level = 5%; *p* = disease prevalence = 50% (maximal estimation); *q* = 1 − *p* (prevalence of having no disease); *d* = margin error or absolute precision = 0.05; $Z_{1-\alpha /2}^{2}$ = 1.96.

Based on the calculation results, the study required a minimum of 385 participants for each diagnosis. Therefore, the minimum sample size required for all the diagnoses (three diseases) to assess the willingness to pay for the catastrophic illness healthcare cost-sharing was 3 × 385 participants (1155 participants). In other words, the study required a minimum of 1155 participants.

### 2.4. Data Collection Tools and Measurements

The data collection was guided by valid structured questionnaires, which were delivered to the participants through direct interviews. There were six parts in the tool, namely socio-demographic and economic characteristics, healthcare-related utilization, healthcare costs, healthcare insurance information, drug-related information, and willingness to pay for NHI cost-sharing (Indonesian version of questionnaire). The questionnaire (English version) was provided for the purpose of this publication. The questionnaire in this study was detailed in Supplementary File S1. The questionnaire was modified from a previous questionnaire on willingness to pay using the contingent valuation method [37,48].

A detailed standard cost-sharing scenario was first explained to all the participants. The participants’ levels of willingness to pay for the prescription drug cost-sharing scheme were then measured by asking questions on whether or not they would be willing to pay a certain amount of premiums for the prescription drug cost-sharing scheme. Double Bounded Dichotomous Choice in the contingent valuation was used to assess the actual amount of premiums that the respondents were willing to pay for the prescription drug cost-sharing scheme.

### 2.5. Data Quality Control

The data collection was done by ten trained pharmacists and supervised by one pharmacist expert. To ensure data quality, the data collection used questionnaires modified from a literature review, validated tools, and the Indonesian Demographic and Health Survey. In addition, the data collection supervision and the collected data validation were performed by investigators and supervisors.

The research assistants were trained by the researcher one week prior to the study. These research assistants were trained not to ask any questions to the participants, so as to avoid data biases. The participants were directed to answer the questionnaire based on their own choices. The training was done several times through online and face-to-face sessions. The supervisors were assigned to assist the research assistants to handle any unexpected conditions—for example, when the participants asked about the legality or objectives of the research. During the study, the research assistants conducted the survey very well.

We also informed the participants about several aspects: their participation was voluntary and could be terminated at any time during the survey; their data were made anonymous, could not be traced back to them, and would be used for academic publication. We then asked the respondents to sign an informed consent form before filling out the WTP survey.

The participants were divided into two groups, namely those from outpatient care and those from inpatient care. The outpatient participants were asked to fill out the questionnaire through direct interviews with the research assistant. Once the participants had filled out the questionnaire, the research assistant checked the completeness of the responses, so that, if unanswered questions were found, the research assistant could directly ask the participants to complete the missing items. On the other hand, the inpatient participants received the questionnaire directly from the research assistant, but they were not accompanied by the research assistant when filling out the questionnaire, because the hospital did not allow any direct meeting with inpatients due to the COVID-19 measures. The research assistant revisited the inpatient participants in the following 1–2 days to take the questionnaire. Unfortunately, some questionnaires were either not returned or were not complete.

### 2.6. Statistical Analysis

Once the data had been checked in terms of completeness, the data were entered into Microsoft Excel and analyzed with the help of SPSS version 24. The characteristics of the participants were described using a descriptive statistics analysis. The statistical relationships between the dependent and independent variables, as well as the factors that affected willingness to pay for the cost-sharing scheme, were identified using a bivariable logistic regression analysis (Table 1). The relationship was reported using a significance of *p* < 0.05 and Adjusted Odds Ratio (AOR) with 95% CI. The model fitness was checked using the Hosmer–Lemeshow test [49].

Regression seeks to insert a set of predictors into a regression equation to explain a variable. The predictors could be inserted into the equation either manually or automatically. SPSS offers several methods to automatically insert the predictors, namely enter, stepwise, forward, and backward. This study used the enter method, i.e., entering all the predictors into the analysis simultaneously. Based on the results of the analysis using SPSS, all the predictors were entered simultaneously.

## 3. Results

### 3.1. Socio-Demographic Characteristics

In this study, 1203 of the 1211 participants completed the interviews, so the response rate was 99.3%. In general, the participants who did not return the questionnaires were inpatient participants. Finally, this study involved a total of 1203 patients as the participants. Most of them (960 participants, or 79.9%) were non-elderly participants. More than half of them were female participants (704 or 58.5%). Most of them were married (85.7%). More than a third of the participants graduated from senior high school (37.5%) and higher education (32.5%); only a few of them did not display a formal education (3.1%). Almost two fifths of the participants had a formal job (39%). More than two fifths of them earned an income of less than IDR 1,500,000 (USD 96.95) per month; some of them also earned no income or had a non-fixed income. Nevertheless, in terms of the (total) household income, more than a third of the participants earned an income > IDR 3,500,000 (USD 226.21). In addition, most of the participants had less than five family members (91.8%) and most of them did not have children under 5 years (91.4%) (Table 2).

### 3.2. Healthcare-Related Factors

The participants were members of the NHI in Indonesia, with various Contributory Health Insurance plans, consisting of class 1, class 2, and class 3. Almost two fifths of the participants chose the class 1 health insurance plan. The participants suffered from illnesses categorized as catastrophic illnesses, including heart disease, cancer, and stroke, receiving either inpatient or outpatient services. Most of the participants in this research were outpatients, with more than one third undergoing outpatient cancer treatment. The study was conducted during the pandemic in 2021, a period when the number of hospitalized patients was lower than usual. Of the 1203 participants, 80.2% had chronic diseases.

The majority of the participants were referred to public tertiary care by their doctors because they had catastrophic illnesses. In the first diagnosis in the primary care setting, the patients who had a catastrophic illness were referred to either secondary or tertiary care. The doctors in the primary care setting conveyed information to the patients and selected the healthcare referral based on the patients’ health condition. Some patients were referred to secondary care; some were directly referred to tertiary care. However, most of them were finally referred to tertiary care.

Among the 1203 respondents, those who chose a healthcare plan in class 1, class 2, and class 3 amounted to 36.9%, 28.8%, and 34.3%, respectively. Most of the participants stated that they were satisfied with the healthcare services (90.4%) and healthcare costs (92.2%). Most of the participants stated that they faced no difficulty in terms of the payment for healthcare costs (95.5%), although, at present, patients on the NHI scheme are not required to make direct payment for the healthcare costs because these costs are paid by the government through the NHI program. However, if the participants had difficulty in making payments for the healthcare costs through the NHI scheme, more than half of the participants chose to make payment by withdrawing money from their savings (Table 3).

### 3.3. Willingness to Pay for Cost-Sharing under National Health Insurance Scheme

**Figure 1:**
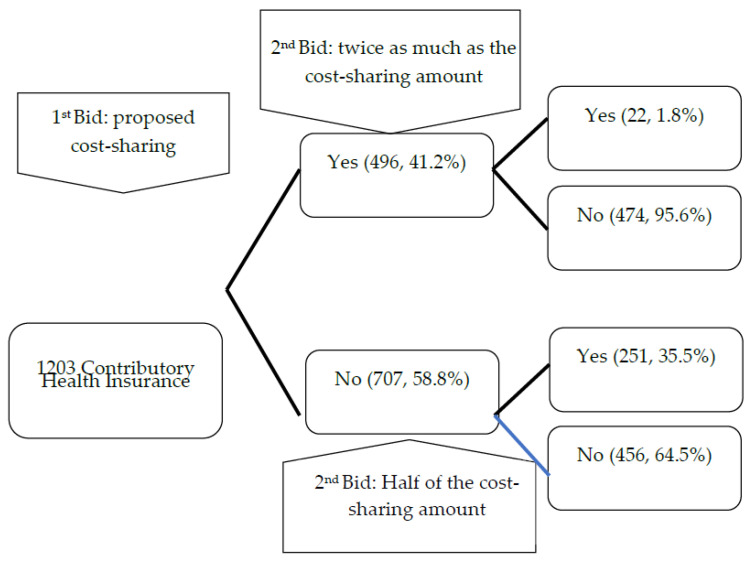
Willingness to pay for cost-sharing under the National Health Insurance scheme.

The contingent valuation method used to obtain the bid amount in this study was the bidding game method, i.e., the respondents were offered a certain amount of money (bid) as the initial/first bid and asked if they were willing to pay this amount. The first bid amount for willingness to pay for cost-sharing under the NHI scheme was determined based on a literature review of the healthcare cost of catastrophic illnesses in Indonesia. The first bid for each of the catastrophic illnesses depended on drug expenditures in Indonesia. The study showed that 496 out of 1203 participants (41.2%) stated their willingness to pay for the health insurance cost-sharing for cancer, heart disease, and stroke. Of these, 22 (1.8%) respondents, who stated their willingness to pay for the first bid amount, also had a willingness to pay twice as much as the first bid amount for each of the three catastrophic illnesses with the highest financing. Of all the participants who were not willing to pay the first bid amount, 251 (35.5%) of them were willing to pay half of the first bid amount (Figure 1).

There were various reasons for which the respondents were not willing to pay for the cost-sharing scheme. Some of the reasons were as follows: 43 (3.6%) participants did not trust the NHI fund management, 9 (0.7%) participants believed that rich citizens should be the ones who paid for the program, 17 (1.4%) participants had already paid the monthly NHI premiums, 624 (51.9%) participants said that the government should bear the responsibility for the health program payment, while 43 (3.6%) of them said that they did not have enough money. In terms of payment of cost-sharing, the majority (946 participants or 78.6%) who expressed a willingness to pay stated that they would prefer that the cost-sharing amount was a percentage of the healthcare cost.

### 3.4. Factors Associated with Willingness to Pay for Cost-Sharing Scheme

The results of the logistic regressions, including the odds ratios and confidence intervals, are displayed in Table 4. The bivariable analysis showed that sex, educational status, family size, occupational status, monthly income, household monthly income, place of treatment, health insurance plan, and whether someone receives an inpatient or outpatient service had a relationship with willingness to pay for the cost-sharing scheme with *p* <0.05. The odds of the female participants’ willingness to pay for cost-sharing decreased by 31% compared with that of the male participants [AOR = 0.69 (0.51, 0.92)]. The odds of willingness to pay for the cost-sharing scheme of households with a larger family size (>five family members) was 1.71 times higher than in those with a small family size [AOR = 1.71 (1.07, 2.73)]. The odds of willingness to pay for the cost-sharing scheme of those who graduated from senior high school was 1.54 higher than those who graduated from higher education [AOR = 1.54 (0.67, 3.55)]. The odds of willingness to pay for the cost-sharing scheme among people with a non-formal occupation was 1.35 times higher than those with a formal occupation [AOR = 1.35 (0.88, 2.07)]. The odds of willingness to pay for the cost-sharing scheme among those whose monthly income ranged from IDR 2,500,000 to 3,500,000 was 1.50 times higher than among those who earned a higher monthly income (>IDR 3,500,000) [AOR = 1.50 (0.87, 2.61)]. Similarly, the odds of willingness to pay for the cost-sharing scheme among the participants whose household monthly income ranged from IDR 2,500,000 to 3,500,000 was 1.47 times higher than among those whose household monthly income was higher (>IDR 3,500,000) [AOR = 1.47 (0.90, 2.39)]. The odds of willingness to pay for the cost-sharing scheme among the participants who went to secondary care (public hospital) was 2.54 times higher than those who went to tertiary care (public hospital) [AOR = 2.54 (1.44, 4.45)]. The odds of willingness to pay for the cost-sharing scheme among the participants who chose a class 3 insurance plan was 1.22 times higher than those whose insurance plan was class 1 or class 2 [AOR = 1.22 (0.87, 1.71)]. Finally, the odds of willingness to pay for the cost-sharing scheme of the female participants were reduced by 31% [AOR = 0.69 (0.51, 0.92)]. In addition, the odds of willingness to pay for the cost-sharing scheme of the outpatients were reduced by 70% compared to that of the inpatients [AOR = 0.23 (0.10, 0.51)] (Table 4).

## 4. Discussion

In the first bid, more than half of the participants were not willing to pay for cost-sharing under the National Health Insurance scheme. Similar to a previous study on WTP for social health insurance (SHI) in Indonesia, the majority of the respondents (87.36%) showed WTP for SHI at the current regulated package. Unfortunately, most respondents (67.58%) did not show any willingness to buy SHI when a cost-sharing system was applied [34]. This study found that 41% of the respondents were willing to pay for the cost-sharing in the first bid. This was in contrast to what was found in a study conducted in Central Nigeria (ranging from N450 (96.6%) to N1200 (72.5%)) [50], and in rural areas in India (70%) [29], where the majority of the respondents were willing to pay for community health insurance. The possible reasons for such differences were the sample size, study period, and socioeconomic status. Most importantly, however, the difference was due to the different health insurance concepts that each of these countries uses. Nevertheless, the second bid amount, which was twice as much as the first bid, significantly decreased the number of participants who were willing to pay for the cost-sharing, i.e., 1.8%. The participants who were asked about the doubled cost-sharing amount were those who were willing to pay for the first bid. In contrast, those who were not willing to pay the first bid amount were then asked about the second bid amount, i.e., half of the first bid amount, and the result showed that only approximately one third of the participants showed WTP for the second bid amount. Cost-sharing in the form of a percentage of the total healthcare cost for catastrophic illnesses (coinsurance) was most preferred by the respondents, compared to cost-sharing with fixed rates (amount), which are applicable for all patients regardless of the healthcare costs of each patient (co-payment). Co-payment is a cost-sharing model that requires patients to pay a fixed out-of-pocket expense per visit, while co-insurance is a cost-sharing model that requires patients to pay a percentage of their healthcare costs as a cost control measure in some healthcare settings. Cost-sharing generally combines several models, including deductibles, co-insurance, co-payments, and limits on participants’ out-of-pocket expenses [13,18,25,51,52].

Many developed countries have implemented cost-sharing in the form of co-insurance or combinations, including France [53], the US (Medicare) [52,54], Japan and Korea [25,55], Belgium [52], China [56], and Canada [57,58,59]. On the other hand, cost-sharing in developing countries is in the form of a percentage of the healthcare costs, including in Ethiopia [60], Pakistan [61], Vietnam [62], Lao [63], Morocco [64], and Thailand [65]. However, the authors did not provide clear information about cost-sharing. Cost-sharing prescriptions were observed in several countries. Australia, England, Finland, Germany, Italy, Taiwan, and China implement a co-payment model for drugs. Meanwhile, countries that implement a mixed cost-sharing model include the US and Canada. Most countries introduced co-payment as the cost-sharing model; only a few used co-insurance as the cost-sharing model, including the US, or in the form of a mixed model, such as in Canada and Switzerland [52].

The factors associated with the willingness to pay for the cost-sharing scheme were examined by multivariate analyses with binary data. Based on the results of this study, healthcare and individual socioeconomic factors were significantly related to the WTP for cost-sharing. Some of the individual socioeconomic factors that affected willingness to pay for cost-sharing under National Health Insurance include sex, educational status, family size, occupational status, and income. The results of the study showed that males tended to have a higher level of willingness to pay for cost-sharing than females. This is supported by a study conducted in Nigeria [38], showing that male respondents had a higher level of willingness to pay for cost-sharing than female respondents. This, however, is different from some studies conducted in Ghana, Senegal, and Mali [66], showing that females had a higher tendency to buy health insurance than males. This is likely because, in Indonesia, the finances of a household are managed by females, so they tend to be more careful in terms of additional spending, i.e., spending for cost-sharing in this case. The participants who had a large family size (>5 family members) had a higher level of WTP for cost-sharing. A previous study showed that a family who had >4 members had a positive association with WTP for SHI [34]; a household with a larger family size had a higher level of WTP compared to that with a smaller family size. This is in accordance with some other studies that were carried out in various countries, including Northeast Kenya [67] and Sudan [14]. One of the reasons behind this might be that those with a large family size will have to spend a greater amount of money when they are in need of health services [67].

The respondents who graduated from senior high school had a higher level of WTP for the NHI cost-sharing scheme than those who graduated from higher education (university education). This is probably because the higher the educational level, the better the awareness of health-related issues, so a person tends to be more critical of policy changes, including the policy concerning cost-sharing in NHI, which has not been introduced previously. However, those with a high school education had a higher level of WTP for the NHI cost-sharing scheme than the respondents who had a lower educational level. Some previous studies mentioned that a higher level of WTP was related to a higher educational level in several countries, including Vietnam [68], Namibia [69], Ghana [70], and Burkina Faso [71]; the WTP for a community-based health insurance scheme was shown to be higher among those who had graduated from primary education than those who had no formal education [4]. This is in line with the findings of a study in Mekelle City, Northern Ethiopia, showing that the higher educational level, the lower the WTP for SHI [37]. On the other hand, a previous study taking place in Bangladesh [72] showed a negative association between educational level and WTP. This might be because health insurance-related knowledge is lacking in Bangladesh, especially among people with a low income [34]. In addition, another study on social policy or community-based health insurance mentioned that educated individuals might engage in better health-seeking behaviors and have a better understanding of the advantages that one can obtain by buying health insurance [4]. Furthermore, the higher the educational level, the better the knowledge about the benefits package, and the higher the level of WTP.

This study showed that those with a non-formal occupation had a higher WTP for the cost-sharing scheme compared to those with a formal occupation. This, however, contradicts the results of research conducted in Northeast India [73] and the Edo State of Nigeria [74]. This is because those with a formal occupation do not have to make a direct payment for the insurance premiums, because the premiums are directly deducted from their salary with a subsidy from the companies in which they work. Therefore, when they were asked about WTP for cost-sharing, most of them believed that the healthcare cost should be the responsibility of the government, so they should not have to incur out-of-pocket expenses for health services. In addition, housewives and students, as unemployed respondents, had a higher level of willingness to pay for cost-sharing compared to those with a formal occupation. This is because those falling within this respondent category might not have had a sufficient understanding of health insurance policies in Indonesia and they did not have to pay a monthly premium directly.

The respondents who had a middle income (IDR 2,500,000–3,500,000) had a higher tendency for WTP for the cost-sharing scheme than those with a lower income. This result is in line with the results of a cross-sectional study in other countries [75]. In general, the different cost-sharing plans are applicable for the citizens who are categorized based on their health status or income to access the same health services or medications [52]. This study showed that the average maximum amount of WTP for the cost-sharing scheme in the form of a percentage was 20% of the healthcare costs or a maximum fixed cost of IDR 500,000. A previous study on WTP for social health insurance in Indonesia showed that most of the participants had willingness to pay for SHI only if the amount was approximately 1.67% of their salary [34]. In Northern Ethiopia, most of the respondents had WTP for SHI if the amount did not exceed 3% of their monthly salary [37].

The respondents who had a higher income (either households or individuals) had a higher tendency to display WTP for cost-sharing than respondents with a lower income. There is theoretically a positive association between WTP and income. The higher the respondents’ household income, the higher the WTP level [23,37]. Similarly, many studies in countries from the low–middle-income group revealed that income had a positive relationship with WTP for social health insurance [37,72] However, the participants in this study whose income was more than IDR 3,500,000 had a lower level of WTP for the cost-sharing scheme. Differences in the economic status of each country might be the reason for this.

In terms of the healthcare factor, the participants who preferred public secondary hospitals as the place of treatment had a higher level of WTP for the National Health Insurance cost-sharing scheme than those who went to a public tertiary hospital or private hospital to seek healthcare services. The NHI financing system in Indonesia, which uses Case Mix INA-CBGs (Indonesia Case Base Group), sets different tariffs among public secondary hospitals, public tertiary hospitals, and private hospitals, in which public secondary hospitals and private hospitals have higher INA-CBG tariffs. The implementation of the INA-CBG payment system is intended to control the healthcare costs in hospital settings. The INA-CBG tariffs are set based on diagnosis and healthcare procedures, despite the number and types of healthcare services accessed. The case-based payment system expectedly helps to improve the quality and efficiency of healthcare services, as well as reducing the payment for over-servicing [10]. Based on the referral system in NHI, the diseases treated in tertiary care are more complicated (severe) than those treated in both primary and secondary care, causing the healthcare costs in tertiary care to be far higher than those for the other two levels of care. In addition, patients who seek healthcare services in tertiary care commonly have poor conditions in relation to the severity of their diseases. This is consistent with the results of this study, in which most of the participants went to tertiary care. In fact, the types of benefits packages and quality of health services affected WTP [34] because the accessibility of a health facility has a significant relationship with health-seeking behavior and the need for cost-sharing [36]. Unlike patients who sought health services in public hospitals, those who sought healthcare services in private hospitals in Indonesia usually had better health conditions and did not suffer from severe illnesses [70]. In a study conducted in Mekelle City, Northern Ethiopia, some of its respondents said that, compared to those in private hospitals, the healthcare costs in public health facilities were fair, but the public health facilities had poor service quality, lacked diagnostic tests, and even faced medication shortages [37].

The participants who chose the class 3 health insurance plan had a higher tendency of WTP for the National Health Insurance cost-sharing scheme than those who chose the other categories of health insurance plans. This is likely because the monthly premium of the class 3 insurance plan is lower than in the other categories. In addition, it is possible to upgrade the insurance plan from class 3 by paying the difference in the INA-CBG tariffs between the two plans. A previous study in Indonesia showed that some respondents decided to upgrade their health insurance plans to obtain higher INA-CBGs tariffs; others were provided with the option for medicine expenditure cost-sharing [10]. Meanwhile, in another study, the fact that the respondents did not have much money and the fact that health was not considered a priority were some of the challenges to WTP [34].

The participants who were hospitalized because of a catastrophic illness had a higher level of willingness to pay for cost-sharing than outpatients. This is because the costs for inpatient services are generally higher than the costs for outpatient services. Eventually, the respondents who suffered from a chronic illness had a higher tendency for WTP for the cost-sharing scheme than those without any chronic illness. This is supported by a study conducted in India [76] and Bangladesh [72]. In addition, risk-averse individuals in general have greater willingness to pay for cost-sharing.

In this study, the reason for which the respondents appeared to be unwilling to pay was that the answers that the respondents wanted to choose were not provided in the questionnaires. If the respondents said yes to all the bid amounts, they were said to have awareness of their answers; the percentage of such was low in this study. When using the contingent valuation, a person has to be aware that the yes or no answers could affect the results’ accuracy [77]. Reasons that the respondents were not willing to pay for the cost-sharing scheme were identified. Some respondents did not trust the NHI fund management; they believed that rich citizens should be responsible for the payment of the program; they had paid monthly NHI premiums, and they could not afford the payment of the program. In addition, another reason mentioned by most of the respondents is that they believed that the government should be responsible for the payment of the healthcare program. It is necessary to note that Indonesia’s public medical system is universally covered, and the healthcare cost is minimal, allowing all members of society, including the very poor, to be able to access the healthcare services. It is common to see people with poor health conditions or older people have access to the public health system.

### Strengths and Limitations of the Study

In terms of strength, the current study used Contingent Valuation Double-Bounded Dichotomous choice, which can be used to minimize response biases. This study can contribute to providing factual insights to the scheme stakeholders, which can be used in the management and organization of the scheme, expectedly improving the acceptability of the scheme among the community members. However, the current study did not reveal the actual amount that the households were willing to pay for the cost-sharing scheme. The respondents who did not have a sufficient understanding of NHI, particularly related to the cost-sharing scheme, probably overestimated their insurance premiums. Such a lack of understanding may lead to the misunderstanding of tariff choices or the forms of cost-sharing. In terms of limitations, this study did not assess the community’s level of knowledge about cost-sharing. A further limitation is that our study was conducted in an area that has the highest level of universal health coverage in Indonesia, and it might not be reflective of the whole of Indonesia.

## 5. Conclusions

This study showed that most of the participants in Yogyakarta did not have willingness to pay for cost-sharing under the National Health Insurance scheme. Sex, educational status, family size, occupational status, monthly income, household monthly income, place of treatment, health insurance plan, and whether someone receives an inpatient or outpatient service were the factors that affected the willingness to pay for the NHI cost-sharing scheme. To increase the community’s interest in the cost-sharing scheme regardless of their socioeconomic status, there are several measures that can be taken. First, the premiums should be adjusted according to socioeconomic factors. Second, socialization at the community level should be conducted to create awareness of the benefits package, the principles of the scheme, as well as overutilization and high healthcare costs for catastrophic illnesses. Third, it is important for the government to pay attention to the cost-sharing model, improve the quality of healthcare services regardless of the fact that the NHI program covers the healthcare program payment, and select and sort appropriate treatments to be included in the cost-sharing scheme so as to not risk the quality of care.

## Figures and Tables

**Table 1:** Variables in logistic regression of factors associated with willingness to pay for cost-sharing.

Variables	Categories	References Category
Age (in years)	<65, ≥65	<65
Sex	Male, female	Male
Current marital status	Single, married, divorce	Single
Educational level	No formal education, elementary school, junior high school, senior high school, higher education	Higher education
Main occupation	Formal occupation, non-formal occupation, unemployed	Formal occupation
Monthly income (IDR)	Uncertain, 0–1,500,000, 1,500,000–2,500,000, 2,500,000–3,500,000, >3,500,000	>3,500,000
Household monthly income (IDR)	Uncertain, 0–1,500,000, 1,500,000–2,500,000, 2,500,000–3,500,000, >3,500,000	>3,500,000
Family size	1–5 > 5	1–5
Family with children under 5 years	Yes, No	No
Having chronic illness	Yes, No	No
Having illness	Inpatient with heart disease, outpatient with heart disease, inpatient with cancer, outpatient with cancer, inpatient with stroke, outpatient with stroke	Inpatient with heart disease
Place of treatment	Public secondary hospital, private secondary hospital, public tertiary hospital	Public secondary hospital
Health insurance plan	1st Class, 2nd Class, 3rd Class	1st Class

Where IDR 1,500,000 = USD 96.95, IDR 2,500,000 = USD 161.58, IDR 3,500,000 = USD 226.21.

**Table 2:** Socio-demographic characteristics of participants (*n* = 1203).

Characteristics	*n* (%)
**Age (in years)**	
Adult (<65)	960 (79.9)
Elderly (≥65)	241 (20.1)
**Sex**	
Male	499 (41.5)
Female	704 (58.5)
**Religion**	
Muslim	1112 (92.5)
Protestant	49 (4.1)
Catholic	41 (3.4)
Hindu	1 (1)
Other	2 (2)
**Current marital status**	
Single	64 (5.3)
Married	1030 (85.7)
Divorced	109 (9.0)
**Educational level**	
No formal education	37 (3.1)
Elementary school	178 (14.8)
Junior high school	147 (12.2)
Senior high school	450 (37.4)
Higher education	391 (32.5)
**Main occupation**	
Formal occupation	467 (39)
Non formal occupation	324 (27)
Unemployed (housewife, student, unemployed)	412 (34)
**Monthly income (IDR)**	
0–1,500,000	551 (45.8)
1,500,000–2,500,000	158 (13.1)
2,500,000–3,500,000	156 (13.0)
>3,500,000	259 (21.5)
Uncertain	79 (6.6)
**Household Monthly income (IDR)**	
0–1,500,000	311 (25.9)
1,500,000–2,500,000	183 (15.2)
2,500,000–3,500,000	194 (16.1)
>3,500,000	426 (35.4)
Uncertain	89 (7.4)
**Family size**	
1–5	1104 (91.8)
>5	99 (8.2)

Where *n* = frequency; % = percentage; IDR 1,500,000 = USD 96.95, IDR 2,500,000 = USD 161.58, IDR 3,500,000 = USD 226.21.

**Table 3:** Participants’ healthcare characteristics (*n* = 1203).

Characteristics	*n* (%)
**Health Insurance Plan**	
Class 1	444 (36.9)
Class 2	346 (28.8)
Class 3	413 (34.3)
**Having illness**	
Inpatient with heart disease	145 (12.1)
Outpatient with heart disease	415 (34.5)
Inpatient with cancer disease	90 (7.5)
Outpatient with cancer disease	460 (38.2)
Inpatient with stroke disease	33 (2.7)
Outpatient with stroke disease	60 (5.0)
**Place of treatment**	
Public tertiary hospital	865 (71.9)
Public secondary hospital	261 (21.7)
Private secondary hospital	77 (6.4)
**Difficulty in making payment for medical expenses**	
No	1149 (95.5)
Yes	54 (4.5)
**If yes, what is the payment alternative? (*n* = 1217)**	
Withdrawing from savings	686 (56.4)
Borrowing	116 (9.5)
Asking for help from relatives or family	328 (26.9)
Doing extra work	24 (2)
Reducing the expenses of other things, e.g., food, tuition fees	6 (0.5)
Other	57 (4.7)

Where *n*= frequency; % = percentage.

**Table 4:** Bivariable logistic regression of factors associated with willingness to pay for cost-sharing under National Health Insurance.

Characteristics	Willingness to Pay for Cost-Sharing		COR (95% CI)	AOR (95% CI)
	Yes, *n* (%)	No, *n* (%)		
**Age (in years)**				
<65 (*n* = 960)	400 (41.7)	560 (58.3)	1	1
≥65 (*n* = 243)	96 (39.5)	147 (60.5)	1.09 (0.82, 1.46)	0.82 (0.58, 1.15)
**Sex**				
Male (499)	195 (39.1)	304 (60.9)	1	1
Female (704)	301 (42.8)	403 (57.2)	0.86 (0.68, 1.09)	0.69 (0.51, 0.92) *
**Current marital status**				
Single (63)	22 (34.9)	41 (65.1)	1	1
Married (1030)	442 (42.9)	588 (57.1)	1.31 (0.68, 2.53)	1.18 (0.57, 2.47)
Divorced (110)	32 (29.1)	78 (70.9)	1.83 (1.19, 2.82)	1.34 (0.82, 2.17)
**Family size**				
1–5 (*n* = 1100)	464 (42.2)	636 (57.8)	1	1
>5 (*n* = 103)	32 (31.1)	71 (68.9)	1.62 (1.05, 2.50) *	1.71 (1.07, 2.73) *
**Educational level**				
Higher education (*n* = 391)	188 (48.1)	203 (51.9)	1	1
Senior high school (*n* = 450)	188 (41.8)	262 (58.2)	2.50 (1.18, 5.31) *	1.54 (0.67, 3.55)
Junior high school (*n* = 147)	43 (29.3)	104 (70.7)	1.94 (0.92, 4.10)	1.52 (0.68, 3.40)
Elementary school *(n* = 178)	67 (37.6)	111 (62.4)	1.12 (0.50, 2.50)	1.11 (0.47, 2.63)
No formal education (*n* = 37)	10 (27)	27 (73)	1.63 (0.74, 3.58)	1.45 (0.63, 3.32)
**Main occupation**				
Formal occupation (*n* = 467)	225 (48.2)	242 (51.8)	1	1
Non-formal occupation (*n* = 324)	130 (40.1)	194 (59.9)	1.79 (1.36, 2.35) ***	1.34 (0.88, 2.07)
Unemployed (*n* = 412)	141 (34.2)	271 (65.8)	1.29 (0.95, 1.74)	1.24 (0.86, 1.80)
**Monthly income (IDR)**				
>3,500,000 (*n* = 259)	143 (55.2)	116 (44.8)	1	1
2,500,000–3,500,000 (*n* = 156)	66 (42.3)	90 (57.7)	2.41 (1.79, 3.243) ***	1.50 (0.87, 2.61)
1,500,000–2,500,000 (*n* = 158)	74 (46.8)	84 (53.2)	1.44 (1.00, 2.05)	1.26 (0.73, 2.16)
0–1,500,000 (*n* = 630)	213 (33.8)	417 (66.2)	1.73 (1.21, 2.46) *	1.42 (0.86, 2.35)
**Household Monthly income (IDR)**				
>3,500,000 (*n* = 427)	231 (54.1)	196 (45.9)	1	1
2,500,000–3,500,000 (*n* = 194)	68 (35.1)	126 (64.9)	2.71 (2.03, 3.60) ***	1.47 (0.90, 2.39)
1,500,000–2,500,000 (*n* = 183)	76 (41.5)	107 (58.5)	1.24 (0.86, 1.78)	0.92 (0.56, 1.50)
0–1,500,000 (*n* = 399)	121 (30.3)	278 (69.7)	1.63 (1.14, 2.35) *	1.17 (0.73, 1.86)
**Having Illness**				
Inpatient with heart disease (*n* = 145)	50 (34.5)	95 (65.5)	1	1
Outpatient with heart disease (*n* = 415)	168 (40.5)	247 (59.5)	0.33 (0.18, 0.61) ***	0.30 (0.16, 0.59) ***
Inpatient with cancer (*n* = 90)	13 (14.4)	77 (85.6)	0.42 (0.24, 0.74) **	0.54 (0.29, 0.97) *
Outpatient with cancer (*n* = 460)	218 (47.4)	242 (52.6)	0.11 (0.05, 0.23) ***	0.11 (0.05, 0.26) ***
Inpatient with stroke (*n* = 33)	10 (30.3)	23 (69.7)	0.56 (0.32, 0.97) *	0.57 (0.31, 1.03)
Outpatient with stroke (60)	37 (61.7)	23 (38.3)	0.27 (0.11, 0,67) **	0.34 (0.13, 0.90) *
**Having chronic illness**				
No (*n* = 238)	129 (54.2)	109 (45.8)	1	1
Yes (*n* = 965)	367 (38.0)	598 (62.0)	1.93 (1.45, 2.57) ***	1.99 (1.44, 2.77) ***
**Place of treatment**				
Public tertiary hospital (*n* = 865)	394 (45.5)	471 (54.5)	1	1
**Public secondary hospital (*n* = 261)**	83 (31.8)	178 (68.2)	2.55 (1.49, 4.36) **	2.54 (1.44, 4.45) **
**Private secondary hospital (*n* = 77)**	19 (24.7)	58 (75.3)	1.42 (0.79, 2.54)	1.73 (0.93, 3.22)
**Health insurance plan**				
Class 1 (*n* = 444)	186 (41.9)	258 (58.1)	1	1
Class 2 (*n* = 346)	164 (47.4)	183 (52.9)	1.32 (1.00, 1.74)	0.91 (0.63, 1.31)
Class 3 (*n* = 413)	146 (35.4)	267 (64.6)	1.65 (1.23, 2.21) **	1.22 (0.87, 1.71)

Where significance at *** *p* < 0.001, ** *p* < 0.01, * *p* < 0.05. IDR 1,500,000 = USD 96.95, IDR 2,500,000 = USD 161.58, IDR 3,500,000 = USD 226.21.

## Data Availability

Not applicable.

## Supplementary Materials

The following supporting information can be downloaded at: https://www.mdpi.com/article/10.3390/ijerph192215017/s1. Supplementary File S1: English version questionnaire.
