# Supplementary material for: Factors Associated with Willingness to Pay for Cost-Sharing under Universal Health Coverage Scheme in Yogyakarta, Indonesia: A Cross-Sectional Survey
Source: Int J Environ Res Public Health. 2022 Nov 15;19(22):15017. doi: 10.3390/ijerph192215017 (PMC9690347; doi:10.3390/ijerph192215017)
Supplement: Supplementary file 1 [file ijerph-19-15017-s001.zip › ijerph-2008858-supplementary.pdf]

Additional File S1: English version questionnaire

Title: Factors Associated with Willingness to Pay for Cost-Sharing under Universal Health Coverage scheme in Yogyakarta, Indonesia

Diagnosis :

Location :

- Outpatient Care ________________ Hospital________________
- Inpatient Care ________________ Class____________ Hospital________________

JKN-KIS/ BPJS-Kesehatan membership as Contributory Health Insurance with class of care:

☐ Class 1 ☐ Class 2 ☐ Class 3

Please choose one response that best describes you by circling it!

Part I: Socio-demographic information		
**No**	**Questions**	**Responses**
1	Date of Birth (month and year of birth)	
2	Gender	1. Male<br>2. Female
3	Religion	1. Islam<br>2. Christianity<br>3. Catholicism<br>4. Hinduism<br>5. Buddhism<br>6. Confucianism,<br>7. Others (specify) ______________
4	Marital Status	1. Unmarried<br>2. Married<br>3. Divorced<br>4. Widowed
5	Highest educational attainment	1. Never attended any education<br>2. Graduated from elementary school<br>3. Graduated from junior high school<br>4. Graduated from senior high school<br>5. Diploma<br>6. University Degree<br>7. Non formal education (certificate)

6	Occupation	1. Civil Servant<br>2. Health Professional<br>3. Teacher/Lecturer<br>4. Private Employee<br>5. Entrepreneur<br>6. Farmer<br>7. Housewife<br>8. Student<br>9. Others (specify) ___________
7	Respondents' net monthly income	IDR
8	Household monthly income (husband and wife)	IDR
9	Family size (including you)	persons
10	Number of children:<br>a. < 5 years old<br>b. 5-21 years old<br>c. >21 years old	a. child(ren)<br>b. child(ren)<br>c. child(ren)
Part II: Healthcare Utilization Information<br>Definition: respondents' response based on their experience of healthcare utilization		
11	Do you or your family member have chronic illness (for example diabetic, hypertension, heart disease, cancer, stroke, asthma, kidney diseases)?	1. Yes<br>2. No
12	How long have you been sick with the current illness?	month(s)
13	How many days have you been absent from work due to the recent episode of illness?	day(s)
14	Where did you get your recent treatment? (You can choose more than one answer)	1. Traditional medicine<br>2. Drug store<br>3. Private healthcare facility<br>4. Public healthcare facility<br>5. Others (specify) ______
15	Why did you go there?	1. It is inexpensive.<br>2. It is not too crowded.<br>3. The illness is self-limiting.<br>4. It is easy to access and convenient.<br>5.I could not afford elsewhere.<br>6. I did not have time to go elsewhere.<br>7. Others (specify)
16	What is your satisfaction level with public or private	1. Very dissatisfied

	healthcare facility service quality?	2. Dissatisfied<br>3. Neutral<br>4. Satisfied<br>5. Very satisfied
17	What is your satisfaction level with the costs of public or private healthcare facilities that are in partnership with BPJS-Kes?	1. Very dissatisfied<br>2. Dissatisfied<br>3. Neutral<br>4. Satisfied<br>5. Very satisfied
Part III: Healthcare Financing Information<br>Definition: respondents' response based on their experience of healthcare financing		
18	How much was your out-of-pocket spending (not covered by BPJS) for the visit, including:<br>a. Round-trip transportation costs<br>b. Accommodation costs outside the hospital<br>c. Meal costs for companion (family members who accompany the patient)<br>d. Illness-related wage/productivity losses<br>e. Doctor visit costs<br>f. Prescription drug costs<br>g. Drug costs apart from prescription drugs (self-medication) or traditional medicine costs<br>h. Medical support services (laboratory, radiology, etc)<br>i. Gratification or payment with goods given to doctor, nurses, or hospital?	a. IDR ____________<br>b. IDR ____________<br>c. IDR ____________<br>d. IDR ____________<br>e. IDR ____________<br>f. IDR ____________<br>g. IDR ____________<br>h. IDR ____________<br>i. IDR ____________
19	How did you pay your healthcare costs?<br>(You can choose more than one answer)	1. BPJS-Kes (free)<br>2. Out-of-pocket spending<br>3. Family<br>4. Private insurance<br>5. Employer<br>6. Others__________
20	How was your financial ability to pay for the healthcare?	1. Affordable<br>2. Unaffordable
21	If paying for the healthcare costs was difficult, how did you finally afford it? (You can choose more than one answer)	1. Withdrawn from savings<br>2. Doing extra work<br>3. Reducing spending on other things, food, school fees<br>4. Borrowing<br>5. Financial assistance from relatives<br>6. Others (specify) ____________
Part IV: Healthcare insurance information<br>Definition: respondents' opinions about national healthcare insurance / BPJS-Kesehatan		

22	Where did you hear about national health insurance/BPJS-Kesehatan?	1. Mass media<br>2. Family<br>3. Friends<br>4. Insurance agent<br>5. Healthcare workers<br>6. Other (specify) ______
23	Do you think national health insurance/BPJS Kesehatan is beneficial?	1. Yes<br>2. No, specify the reason(s)____________________
24	If yes, which of the following benefits do you expect from healthcare insurance? (You can choose more than one answer)	1. avoid unexpected health costs<br>2. help others who could not afford their healthcare costs<br>3. improve the quality of healthcare services<br>4. receive timely care in times of sickness<br>5. Others (specify)______
25	What kinds of healthcare insurance do you have to cover your healthcare costs except BPJS Kesehatan?	1. Private insurance<br>2. Employment-based health insurance apart from BPJS-Kesehatan<br>3. Others (specify)____________
26	How many family members (including you) that are covered by your healthcare insurance?	___________ persons
27	How much is your monthly premium for the healthcare insurance for the whole family members?	IDR
Part V: Drug-related information<br>Definition: respondents' opinions about prescription drugs		
28	As far as you can remember, how many prescriptions have you received during the last episode of illness?	_________prescriptions
29	How many prescription drugs (total number of drugs) have you received during the last episode of illness?	__________drugs
30	Did you get all the drugs prescribed to you in the last episode of illness?	1. Yes<br>2. No
31	If no, how many prescription drugs have you got with out-of-pocket spending (not covered by BPJS-Kesehatan) in the last episode of illness?	

32	How many over-the-counter drugs have you got with out-of-pocket spending in the last episode of illness?	

Part VI. Willingness to Pay Scenario

In the following, there are three health financing scenarios. Please identify which health financing scenario you prefer.

<u>Scenario A: No insurance</u><br>You will have to make full payment for each visit to any healthcare facility and for any prescription drugs given to you and your family. If you cannot afford to pay, you will not receive any services. A service is provided with a payment. No exception (free services). The total annual healthcare costs will depend on the number of family members who get sick and visit healthcare facilities.
<u>Scenario B: Mandatory healthcare insurance</u><br>All citizens are obliged to pay a monthly premium for healthcare financing. All Indonesian citizens are obliged to enroll, no exceptions. The premium is a certain amount of money that is paid directly every month, or a portion directly deducted from the monthly salary as determined by the government. All citizens who pay monthly premiums are entitled to receive the same coverage for the risks of illness (the amount of the premiums remain the same regardless of the illnesses).
<u>Scenario C. Mandatory healthcare insurance with cost-sharing</u><br>All citizens are obliged to pay a monthly premium for healthcare financing. All Indonesian citizens are obliged to enroll, no exceptions. The premium is a certain amount of money that is paid directly every month, or a portion directly deducted from the monthly salary as determined by the government. All citizens who pay monthly premiums are entitled to receive healthcare service at the nearest healthcare facilities, with drug prescription cost-sharing for certain illnesses. The cost-sharing amount is determined by the government.

Part V: Health financing scenario
Definition: respondents' opinions about health insurance financing scenario, particularly related to cost sharing

**No**	**Questions**	**Responses**
33	Which scenario do you prefer?	1. A<br>2. B<br>3. C
34	Do you think the government must specify certain illnesses in scenario C?	1. Yes<br>2. No
35	If most people prefer scenario C and the system is to be implemented, are you still willing to enroll in BPJS-Kesehatan?	1. Yes<br>2. No

36	If **Yes** for Question 35, what is your reason? (You can choose more than one answer)	1. I support the government policy.<br>2. I have to help others who cannot afford their healthcare costs.<br>3. I often have health problems.<br>4. It gives me peace of mind in times of sickness.<br>5. Others (specify) ________________
	If **No** for Question 35, what is your reason? (You can choose more than one answer)	1. I do not trust the government program.<br>2. I do not trust the insurance scheme.<br>3. I cannot afford the premium.<br>4. The insurance package does not cover all healthcare services<br>5. I prefer to pay the excess amount.<br>6. Public healthcare facilities have poor healthcare service quality.<br>7. Others (specify) __________________
37	If scenario C (cost-sharing) is to be implemented, which one do you prefer?<br><br>*If you **prefer number 1**, please proceed to Question 38A (**choose according to your disease diagnosis**)<br>**If you **prefer number 2**, please proceed to Question 38B (**choose according to your disease diagnosis**)	1. Cost-sharing in the form of **percentage** of the total healthcare costs<br>2. Cost-sharing in the form of certain fixed **amount of money (IDR)** (not depending on prescription drug costs)
38A	Do you have willingness to pay if the cost-sharing is **40%** of the **total healthcare costs for your cancer inpatient services**?	1. Yes<br>2. No
	Do you have willingness to pay if the cost-sharing is **20%** of the **total healthcare costs for your stroke inpatient services**?	
	Do you have willingness to pay if the cost-sharing is **30%** of the **total healthcare costs for your heart disease inpatient services**?	
	Do you have willingness to pay if the cost-sharing is **35%** of the **total healthcare costs for your cancer outpatient**	

	**services**?	
	Do you have willingness to pay if the cost-sharing is **75%** of the **total healthcare costs for your stroke outpatient services**?	
	Do you have willingness to pay if the cost-sharing is **75%** of the **total healthcare costs for your heart disease outpatient services**?	
38B	Do you have willingness to pay if the cost-sharing is IDR **3,500,000** for the **healthcare cost of your cancer inpatient services**?	1. Yes<br>2. No
	Do you have willingness to pay if the cost-sharing is IDR **2,000,000** for the **healthcare cost of your stroke inpatient services**?	
	Do you have willingness to pay if the cost-sharing is IDR **1,500,000** for the **healthcare cost of your heart disease inpatient services**?	
	Do you have willingness to pay if the cost-sharing is IDR **3,000,000** for the **healthcare cost of your cancer outpatient services**?	
	Do you have willingness to pay if the cost-sharing is IDR **500,000** for the **healthcare cost of your stroke outpatient services**?	
	Do you have willingness to pay if the cost-sharing is IDR **500,000** for the **healthcare cost of your heart disease outpatient services**?	
39	If **Yes** for Question 38A or 38B, are you willing to pay **double the amount** of Question 38A or 38B?	1. Yes<br>2. No
	If **No** for Question 38A or 38B, are you willing to pay **half the amount** of Question 38A or 38B?	1. Yes<br>2. No
40	What is **the maximum** percentage or amount (IDR) of the prescription drug costs that you are willing to pay?	__________% of the total healthcare costs<br><br>or<br><br>IDR __________

41	If **0% or IDR 0** for Question 40, what is your reason?	1. I doubt the fund management.<br>2. The government should be responsible for the payment of such program.<br>3. I do not have enough money.<br>4. Other rich society members should pay for the program.<br>5. Others (specify)<br>________________

Thank you for your participation.
